# Factors that regulate embryonic gustatory development

**DOI:** 10.1186/1471-2202-8-S3-S4

**Published:** 2007-09-18

**Authors:** Robin F Krimm

**Affiliations:** 1 Department of Anatomical Sciences and Neurobiology, University of Louisville School of Medicine, Louisville, KY, USA

## Abstract

Numerous molecular factors orchestrate the development of the peripheral taste system. The unique anatomy/function of the taste system makes this system ideal for understanding the mechanisms by which these factors function; yet the taste system is underutilized for this role. This review focuses on some of the many factors that are known to regulate gustatory development, and discusses a few topics where more work is needed. Some attention is given to factors that regulate epibranchial placode formation, since gustatory neurons are thought to be primarily derived from this region. Epibranchial placodes appear to arise from a pan-placodal region and a number of regulatory factors control the differentiation of individual placodes. Gustatory neuron differentiation is regulated by a series of transcription factors and perhaps bone morphongenic proteins (BMP). As neurons differentiate, they also proliferate such that their numbers exceed those in the adult, and this is followed by developmental death. Some of these cell-cycling events are regulated by neurotrophins. After gustatory neurons become post-mitotic, axon outgrowth occurs. Axons are guided by multiple chemoattractive and chemorepulsive factors, including semaphorins, to the tongue epithelium. Brain derived neurotrophic factor (BDNF), functions as a targeting factor in the final stages of axon guidance and is required for gustatory axons to find and innervate taste epithelium. Numerous factors are involved in the development of gustatory papillae including Sox-2, Sonic hedge hog and Wnt-β-catenin signaling. It is likely that just as many factors regulate taste bud differentiation; however, these factors have not yet been identified. Studies examining the molecular factors that regulate terminal field formation in the nucleus of the solitary tract are also lacking. However, it is possible that some of the factors that regulate geniculate ganglion development, outgrowth, guidance and targeting of peripheral axons may have the same functions in the gustatory CNS.

## Introduction

The unique morphology of the taste system makes it ideal for the study of the molecular factors regulating sensory development. For example, mammalian lingual taste buds develop within specialized structures called papillae, which are located in a specific spatial array. These fungiform papillae provide discrete targets for innervating neurons, making this system ideal for examining factors regulating neuronal targeting during development. It is also the case that gustatory neurons project to specific regions of the oral cavity that contain taste buds (tongue and palate); therefore, unlike nociceptors or mechanoreceptors they can be identified with retrograde tracers. In addition many of the same factors that regulate gustatory development (like BDNF) may also be important for CNS development and/or function. In spite of the multiple advantages that the taste system provides to a general understanding of developmental neurobiology, it has not been widely studied. Some important factors regulating taste system development have been identified. However, the majority of these regulatory factors and the mechanisms by which they function are largely unknown.

The goal of this article is to provide an overview of some of the molecular factors that influence the embryonic development of the rodent gustatory system. A general timeline of gustatory development is provided in Table 1. Processes for which there is little understanding of the molecular factors involved will also be mentioned. This review will focus on the development of the primary sensory neurons in the geniculate and petrosal ganglia. Specifically, the formation of the epibranchial placodes, neuronal differentiation within gustatory ganglia, cell cycle influences on gustatory ganglion development, and axonal outgrowth and guidance will be discussed. Next, the factors regulating the development and innervation of the peripheral target of gustatory neurons, the fungiform papillae and taste buds will be reviewed. This article will then conclude with a brief discussion of the regulation of central projections of these neurons into the rostral nucleus of the solitary tract (NST) and the formation of postsynaptic NST neurons. This discussion is not intended to provide a complete review of gustatory development as there are numerous review articles describing the functional and morphological development of the taste system in other taste bud containing regions, across species, in more detail [1-8]. Please refer to those articles for a more complete understanding of taste system development.

**Table 1:** Timeline of morphological development in the taste system.

Gustatory ganglion	Placode formation	Placode delamination migration	Initial axon outgrowth	Peak cell production	Axons reach tongue	Peak cell death	Target innervation
Mouse	**E8.5**	**E9.5**	E9.5	E10.5	**E12**	E14.5	**E14-15**
Rat	**E9.5-10**	E11	**E11.5**	**E12.5**	**E13.5**	**E16.5**	**E16.5**

Tongue and taste buds	Tongue	Fungiform papillae (placode) (SEM)	Full no. of fungiform papillae	Taste bud differentiation begins	Taste pores	Full no. of vallate taste buds	

Mouse	**E12**	**E13-13.5**	**E14.5**	**E16.5**	**postnatal**	**adult**	
Rat	**E13.5**	**E14.5-15.5**	E16.5	**E20.5**	**postnatal**	**adult**	

A general timetable of major morphological changes is provided for rats and mice. The first sperm/plug positive day is considered day 0.5. Mice typically develop two days earlier than rats. The bold time points have been determined experimentally, non-bold numbers are estimated values based on this two-day difference.

## Development of the geniculate and petrosal ganglia

**Figure 1:**
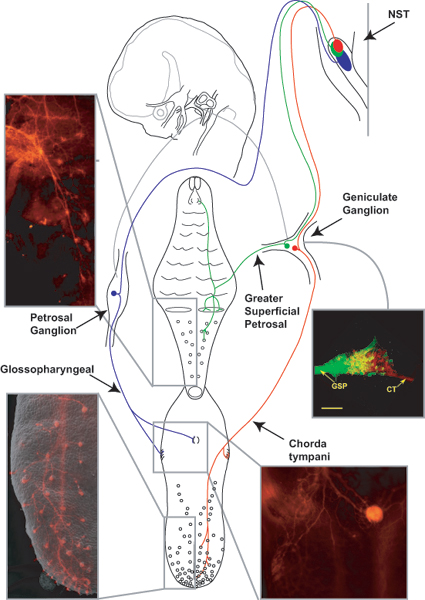
**An overview of the basic neuroanatomy of the gustatory system.** A cartoon of geniculate neurons innervating the tongue (red) and the palate (green) and petrosal neurons innervating the tongue (blue) are shown innervating peripheral taste bud containing regions and the rostral nucleus of the solitary tract (NST). On the tongue, taste buds are located in fungiform papillae, foliate papillae, and circumvallate papillae (CV). The palate has taste buds on the nasoincisor papilla/ducts (NID) and on the soft palate (circles). Photomicrographs of innervation patterns in the tongue and in the palate at E16.5 are shown next to the appropriate regions. An overlay image of two geniculate ganglia (E14.5) is also shown; one ganglia following DiI-label to the palate was pseudo-colored green, the other following DiI-labeling of the tongue remains red. These two ganglia images were anatomically aligned and superimposed using Adobe Photoshop.

Primary gustatory neurons are typical pseudo-unipolar sensory neurons that relay information from the taste buds to CNS neurons located in the rostral portion of the nucleus of the solitary tract (NST; Figure 1). In rodents, the taste buds these neurons innervate are located in specialized structures called papillae (fungiform, foliate and circumvallate) on the tongue, in the nasoincisive papilla and eminences on the soft palate. Sensory neurons of the geniculate ganglion innervate taste buds on the front of the tongue and on the palate via the chorda tympani and the greater superficial petrosal nerves, respectively. The geniculate ganglion also provides somatosensory innervation to the outer ear. Neurons of the petrosal ganglion, on the other hand, innervate taste buds within the circumvallate and foliate papillae via the glossopharyngeal nerve. The petrosal ganglion also contains other chemoreceptors such as baroreceptor neurons, which innervate regions of the cardiac outflow tract and monitor blood pressure. Both the petrosal and the geniculate ganglia arise primarily from epibranchial placodes, but probably also include some cells from neural crest [9]. The unproven dogma is that epibranchial placodes, not neural crest, gives rise to the gustatory portion of these ganglia. Therefore, we will begin by discussing the development of the epibranchial placodes.

### Induction and formation of epibranchial placodes

Epibranchial placodes are transient ectodermal thickenings. The first epibranchial placode differentiates to form the geniculate ganglion, and the second forms the petrosal ganglion. There is accumulating evidence that all cranial placodes, including the epibranchial placodes, arise from a common placodal primordium [10]. Early on, the *Six1/2 *and *Six4/5 *subfamilies and the *Eya *family of transcription factors are expressed in the horseshoe-shaped area of pre-placodal region. Disruption of any of these genes disrupts the development of multiple placodes. Therefore, these genes may regulate multiple placodal properties. The epibranchial placodes are among those affected by mutations in the *Eya *family. In *Eya1 *null mice, the development of the geniculate and petrosal ganglia is completely blocked [11], and both ganglia fail to express important downstream differentiation factors. The early arrest of the differentiation program in these ganglia causes the cells to undergo apoptosis. Six1 interacts with Eya1 and is not expressed in *Eya1*^-/- ^mice. *Six1 *gene mutations, which have less severe effects than *Eya1 *mutations, result in the absence of the geniculate ganglion and a partial loss of the petrosal ganglion. Therefore, members of the *Six1/2 *and *Six4/5 *subfamilies and the *Eya *family are required for geniculate and petrosal ganglion development and may be important for conferring a pan-placodal fate in developing ectoderm.

Induction of individual placodes from the common placodal primordium is likely to be a complex multistage process [10]. Following expression of genes in the *Six *and *Eya *families, other transcription factors are expressed in multiple placodes in partially overlapping patterns. These factors likely define subsets of placodes. One set of factors that could confer placodal identity are the *Pax *genes. *Pax2 *and *Pax8 *are expressed in the posterior placodal region, where the epibranchial and otic placodes are derived [10]. Pax2 may regulate epibranchial neuron identity [12] and is important for otic placode development [10]. Because Pax2 expression is more restricted than the pan-placodally expressed genes, it may be important for conferring placodal identity. However, since Pax2 expression is present in both the epibranchial and otic placodes, it must cooperate with other factors to specify epibranchial placode identity.

In addition to transcription factors mentioned above, signals arising from the pharyngeal pouch are also important for epibranchial placode formation [13]. Included among these signals are members of the bone morphogenic protein family (BMP). The pharyngeal pouch endoderm expresses BMP7, which has been shown to induce the formation of epibranchial placodes when ectopically expressed in a chick embryo. Interestingly, removal of functional BMP7 in mice causes deficits that are restricted to the developing eye and kidney [14,15]. Therefore, it is possible that BMP7 is not important for epibranchial placode formation in mice or more likely, that another BMP family member functions redundantly with BMP7 to induce epibranchial placode formation [15,16]. BMP7 expression is unaffected by absence of *Eya1 *[11]. Thus, BMP signalling is independent of early pan-placodal transcription factors like *Eya1*.

### Neuronal specification within the epibranchial placodes

Cells of the epibranchial placodes differentiate into neuroblasts and subsequently delaminate (occurring on E9 in mice), migrate, and coalesce to form the geniculate and petrosal ganglia [17]. In general, placodal neuronal differentiation is under the control of the basic-helix-loop-helix (bHLH) transcription factors, which are related to the Drosophila *atonal *and *achaete-scute *genes [10]. One such bHLH transcription factor is neurogenin 2 (*Ngn2*). *Ngn2 *expression is dependent on *Eya1 *[11] and strong *Ngn2 *expression is first observed in the geniculate placode at E8.5 and in the petrosal placode at E9.0 [18], while the related factor *Ngn1 *is only weakly expressed. The placodal neuroblasts that give rise to the geniculate and petrosal ganglia fail to delaminate and migrate in the absence of *Ngn2 *[19]. These neuroblasts also fail to express neuron differentiation markers (e.g., neurofilaments), indicating that *Ngn2 *regulates neuronal fate determination for the geniculate and petrosal ganglia. *Ngn1 *does not appear to share this function as the development of these ganglia is unaffected by *Ngn1 *knockout [18]. However, *Ngn1 *is required for the development of the trigeminal and otic ganglia [18].

In addition to *Ngn2*, the homeodomain transcription factors, *Phox2a *and *Phox2b*, regulate pan-neuronal fate in the geniculate and petrosal ganglia. *Phox2a *expression precedes *Phox2b *expression in the geniculate and petrosal ganglia. Accordingly, *Phox2a *expression is dependent on *Eya1*, but is independent of *Ngn2*, and *Phox2b *expression is dependent on *Ngn2 *[11,19]. Studies of *Phox2a *null animals reveal that the geniculate and the petrosal ganglia atrophy in the absence of this transcription factor [20]. Moreover, in the absence of *Phox2b*, the geniculate and petrosal ganglia degenerate [21]. Thus, both *Phox2 *genes are clearly necessary for the continued differentiation of the geniculate and petrosal ganglia.

### Differentiation of gustatory specific cell traits

The factors discussed so far confer a neuronal fate to epibranchial placode derived neurons, most of which are visceral sensory neurons. Since development of the trigeminal ganglion is regulated by a different set of factors, it could be argued that these factors specifically regulate visceral sensory fate rather than pan-neuronal fate. However, the visceral sensory neurons of the geniculate and petrosal ganglia are made up of several neuronal subpopulations. For example, in addition to gustatory neurons, the petrosal ganglion also contains baroreceptive neurons. Moreover, the gustatory neuron population can be divided further based on the taste bud population they innervate and their physiological response characteristics. Currently, nothing is known about how the differentiation of specific neuron sub-phenotypes in the geniculate and petrosal ganglia is regulated. However, the following possibilities are likely.

Coordinated expression of an, as yet, unidentified family of transcription factors may confer specialized gustatory neuron phenotypes. There is precedence for this scenario in other sensory ganglia like the dorsal root ganglia (DRG). In the DRG, the transcription factor *Runx1 *regulates the development of channels and receptors that transduce pain. A related factor, *Runx3*, regulates proprioceptive neuron differentiation [22,23]. A similar mechanism may underlie regulation of visceral neuron subtype differentiation in the geniculate and petrosal ganglia. Large-scale expression mapping [24] has been used to identify transcription factors regulating somatosensory neuron subtypes. A similar approach could prove useful in the identification of transcription factors expressed in and capable of regulating the differentiation of geniculate and petrosal subpopulations.

The differentiation of specific neuron sub-phenotypes may also be regulated by the same factors that act early in the general differentiation of neurons. It is possible that the expression of these factors becomes restricted to specific subpopulations of neurons within the geniculate and petrosal ganglia as development proceeds. These factors may then regulate differentiation of neuronal subpopulations. There is evidence to support this model of neuron subtype differentiation. For example, *Phox2 *genes, which play a role in early neuronal differentiation, have been shown to impart cell-specific traits to geniculate and petrosal neurons. During embryonic development, both the geniculate and petrosal ganglia temporarily adopt a noradrenergic phenotype, which requires *Phox2a *expression [20]. *Phox2a *is also required for the development of another cellular trait, the expression of a receptor subunit (c-Ret) for the glial-derived family of neurotrophins. Later in embryonic development (E16.5), co expression of *Phox2a *and *Phox2b *defines a population of neurons in the petrosal ganglion that expresses the dopamine-synthesizing enzyme, tyrosine hydroxylase (TH), in response to depolarizing stimuli [25]. These neurons are the chemoafferents that innervate the carotid body and are important for regulating breathing. Unfortunately, none of the neuronal traits examined thus far are relevant to a taste neuron phenotype, and there is no evidence to support a role for *Phox2 *genes in the specific regulation of taste neuron differentiation.

**Figure 2:**
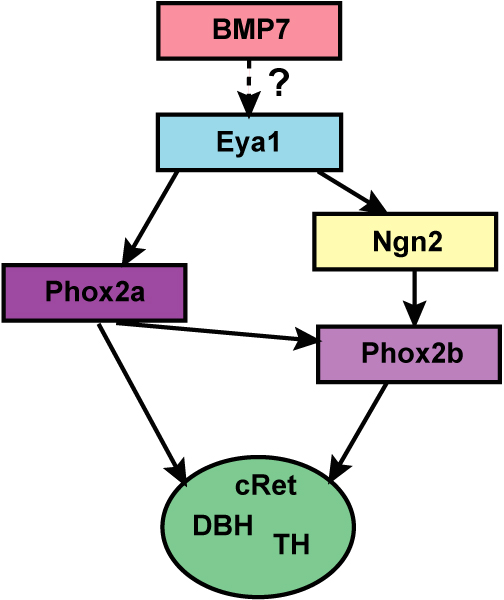
**During development, the early differentiation of the geniculate and nodose ganglia is regulated by a series of transcription factors and signals from the pharyngeal pouch endoderm.** The *Six *and *Eya *families of transcription factors are important for the development of multiple placodes, including the epibranchial placodes, from a single, common placode. In this pan-placodal area, Pax2 expression demarcates a region that forms the epibranchial and otic placodes. Signals from the pharyngeal pouch endoderm, like members of the bone morphogenic protein family (BMP), are required to induce epibranchial placode formation. Also, neurogenin 2 (Ngn2) and Phox2a signaling are important for neuronal differentiation within the placodes. Both are dependent on *Eya1*, but independent of one other. *Phox2b *is dependent on both *Phox2a *and *Ngn2*. *Phox2 genes *may be important for general neuronal differentiation as well as for differentiation of neuron subtypes. We propose that an unidentified factor(s) regulates the differentiation of gustatory phenotype and subtypes within these ganglia.

Factors other than transcription factors may also regulate gustatory sub-phenotype. For example, a group of growth factors called neurotrophins, which influence multiple aspects of neuron development, can also influence neuronal differentiation [26,27]. Neurotrophins have been shown to regulate distinctive neurophysiological properties of geniculate neurons *in vitro *[28]. This finding indicates that neurotrophins may regulate the functional differentiation of gustatory neurons. The neurotrophins will be discussed in more detail in the following section. In summary, gustatory neuron development undoubtedly requires a hierarchical signalling cascade (Figure 2), most of which have not yet been identified.

### Gustatory ganglia cell cycle dynamics

Following migration, a transit-amplifying population of neuronally committed cells (i.e., neuronal precursors) continue to proliferate, resulting in ganglion expansion [29]. A balance between the number of cells that are initially born in the ganglia, the number that differentiate into neurons, and the number that die determines the final number of neurons within a ganglion. For the geniculate ganglion, neuron production peaks at approximately E12 in rats [29], which is roughly equivalent to E10 in mice. However, proliferation occurs over a fairly prolonged period. It is not known how or if terminal mitosis relates to neuronal phenotype. For example, are gustatory neurons of the geniculate generated before, after, or at the same time as somatosensory neurons innervating the external ear? Are palatal and tongue gustatory neurons generated at different times? These questions can be addressed by techniques that allow the precise temporal discrimination of when terminal mitosis occurs [30]. Knowing if subpopulations are generated at the same or different times may provide incites into the factors that regulate their specification as well as those that control cell proliferation for these neurons. It is also not known how many stem cells contribute the formation of the gustatory ganglia or whether or not clonally related precursors contribute to multiple or only one neuronal subpopulation.

Typically, during development, neurons are overproduced and ganglia undergo a period of developmental cell death. In the geniculate ganglion, the total number of neurons remains fairly constant across embryonic age, indicating that new neurons are differentiating at the same rate as others are dying [31]. Neuronal death reaches its peak at E16.5 in rat, which approximates E14.5 in mice. It is at this embryonic age that gustatory fibers first penetrate the epithelial surface of fungiform papillae to form neural buds [32-34]. The finding that geniculate neuron death peaks during target innervation is consistent with the possibility that factors produced by fungiform papillae regulate neuron survival.

One set of factors, produced by neuronal targets and known to regulate sensory neuron survival, are the neurotrophins [35]. The neurotrophins are a group of structurally and functionally related growth factors. There are four members of the neurotrophin family in mammals: nerve growth factor (NGF), brain-derived neurotrophic factor (BDNF), neurotrophin-3 (NT3), and neurotrophin-4 (NT4/5). In addition to their classic target-derived role, neurotrophins are produced in and near sensory ganglion where they influence neuronal cell cycle kinetics and differentiation [27,36-41].

**Figure 3:**
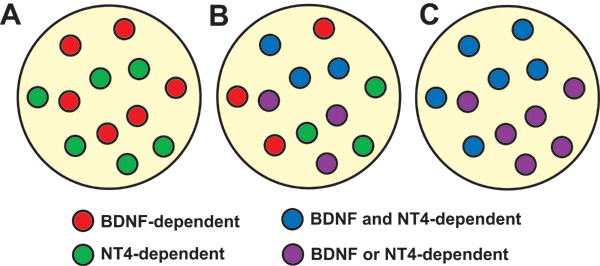
***Bdnf***^-/- ^**and*****Ntf5***^-/- ^**mice lose 50% of geniculate/petrosal and nodose neurons**. Mice lacking both BDNF and NT4/5 lose almost all of the neurons in these ganglia. At least two different scenarios could explain these findings. Two separate subpopulations could exist. One that is BDNF-dependent and one is that is NT4/5-dependent (A). In this case, BDNF dependent neurons would be lost in *Bdnf*^-/- ^mice and NT4/5-dependent neurons are lost in *Ntf5*^-/- ^mice. Alternatively, one subpopulation of geniculate/petrosal/nodose neurons may be dependent on both BDNF and NT4/5 (C) such that removal of either neurotrophin would result in death. These two possibilities are not mutually exclusive. That is, all four types of neuron dependencies could be present in the same ganglion (B).

BDNF, NT3, and NT4/5 are important regulators of geniculate and nodose/petrosal neuron number. *Bdnf*^-/- ^and *Ntf5*^-/- ^(NT4/5 is encoded by the Ntf5 gene) mice lose approximately half of their geniculate ganglion and nodose/petrosal ganglion complex during development. Hybrid *Bdnf*^-/-^*/Ntf5*^-/- ^animals lose 90–94% of their geniculate and nodose/petrosal neurons [42-45]. These findings indicate that there are multiple subpopulations in the geniculate and petrosal ganglia that differ in their neurotrophic factor dependence. At least two different scenarios of neurotrophic factor dependence in these subpopulations could account for these findings (Figure 3). In the first scenario, one subpopulation is BDNF-dependent and another is NT4/5-dependent. Accordingly, BDNF dependent neurons are lost in *Bdnf*^-/- ^mice, and NT4/5-dependent neurons are lost in *Ntf5*^-/- ^mice. An alternative, but not mutually exclusive possibility, is that one subpopulation of geniculate/petrosal/nodose neurons may be dependent on both BDNF and NT4/5. In this case, removal of either neurotrophin would result in the death of this subpopulation. The other subpopulation would require either BDNF or NT4/5 for survival and are only lost when both neurotrophins are removed. If both scenarios are correct, four types of neuron dependencies could be present in the same ganglion and the removal of both neurotrophins would lead to the loss of all subpopulations in question. It is not clear how these differing dependencies will be sorted out. Although some information might be gained the neurotrophic factor dependencies of each gustatory subpopulation is determined. In addition, these scenarios do not account for the pro-survival effects of NT3. Mice lacking NT3 (encoded by the *Ntf3 *gene) lose about 47% of the neurons in the geniculate ganglion and 44% in the nodose/petrosal ganglion complex [44]. With this in mind, it is clear that some overlap in the neurotrophin dependencies of these neurons must exist.

Taste buds require innervation for their maintenance [46-48]. Consequently, fungiform papillae and taste buds are lost in *Bdnf*^-/-^and *Ntf5*^-/- ^mice, showing that both BDNF and NT4/5 support gustatory neurons of the geniculate [49-52]. On the other hand, circumvallate taste buds are lost in only in *Bdnf*^-/-^mice [49,51,53]. Thus, BDNF, not NT4/5, is required for petrosal gustatory neuron survival. No fungiform papillae are lost in *Ntf3*^-/- ^mice [51]. However, *Bdnf*^-/-^*/Ntf3*^-/- ^animals exhibit more taste bud loss than *Bdnf*^-/- ^animals [54], leaving it unclear whether NT3 regulates gustatory neuron number and/or taste buds. It is also unclear whether taste buds are lost solely because of a loss of neurons in neurotrophin mutants. That is, neurotrophins produced in the tongue may have autocrine or paracrine effects on taste bud development or maintenance.

The source of the neurotrophins influencing gustatory ganglion development is presently unknown. BDNF and NT3 are expressed in gustatory papillae, taste buds, geniculate ganglia, and in the rostral nucleus of the solitary tract (NTS) [51,55-61]. Although less well studied, NT4/5 expression has been observed in the tongue, taste buds, and in neurons in the geniculate ganglion [62-64]. The presence of neurotrophins in these various locations within the taste system, suggests that they would be capable of regulating cell cycle events at a wide range of embryonic time points.

**Figure 4:**
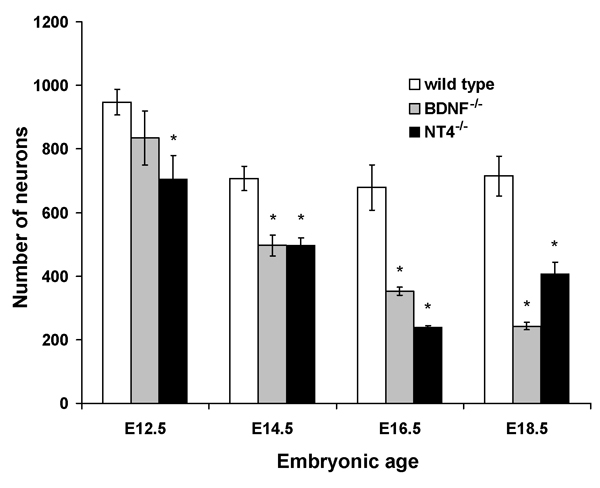
**Neurons are lost throughout embryonic development in *Bdnf*^-/- ^and *Ntf5*^-/- ^mice**. At E12.5, the geniculate ganglion is still fused with the vestibular-cochlear ganglion, which explains the greater number of neurons in wild type mice at this age. Neurons are lost by E12.5 in *Ntf5*^-/- ^mice, indicating that NT4/5-dependency begins earlier in embryonic development than does BDNF-dependency. In *Bdnf*^-/- ^mice, neurons are first lost between E12.5 and E14.5. Losses continue to be greater in these animals, compared to wild type, throughout the remainder of embryonic development.

In other sensory ganglia, the timing of cell loss in mice lacking neurotrophins has been an important indicator of the specific cell cycle event influenced by neurotrophins [37,38,44]. The earliest loss of neurons in the nodose/petrosal complex is observed in *Ntf3*^-/-^mice by E12.5. In *Ntf5*^-/- ^mice, loss of these neurons occurs by E13.5 and in *Bdnf*^-/- ^mice loss occurs by E14.5 [65]. These results have led to the conclusion that neurons require NT3 and NT4/5 for survival during early ganglion development, before target innervation occurs, and that they become dependent on BDNF once they innervate their targets. While this is a plausible explanation of these data, it is not clear whether NT3 and NT4/5 continue to support nodose/petrosal neuron survival after E14.5, which would argue against a switch in dependency. Our laboratory has recently examined the timing of neuron loss in the geniculate ganglion in mice lacking neurotrophins (Figure 4) [66]. We have observed that geniculate neurons are first lost in *Bdnf*^-/- ^mice from E12.5 to E14.5, which is just before or at the onset of target innervation. Geniculate neurons continue to be lost at a greater rate in these animals, compared to wild-type mice, through E18.5 of development and thus, well after target innervation. In *Ntf5*^-/- ^mice, the initial loss of geniculate neurons occurred before E12.5, preceding the loss of the nodose/petrosal complex in these animals. These results are consistent with an early role for NT4/5 in gustatory ganglion development. Interestingly, a second set of neurons was also lost between E14.5 and E16.5. These observations reveal that NT4/5 regulates neuron loss at two distinctive time points; one before target innervation and one after. After E16.5, neurons are no longer lost but are added to the geniculate ganglion in *Ntf5*^-/- ^mice. Together, these findings indicate that BDNF regulates geniculate neuron survival for a prolonged embryonic period that begins during target innervation while NT4/5 regulates neuron number at several distinct stages. These findings argue against a simple switch in dependence from NT4/5 early in development to BDNF later. It is more probable that each neurotrophin has multiple roles and may utilize multiple mechanisms for regulating geniculate ganglion cell cycle dynamics.

Very little data is available regarding the mechanisms by which BDNF and NT4/5 regulate the proliferation and death of neurons in the petrosal and geniculate ganglia. Neuron loss in the petrosal ganglion of *Bdnf*^-/- ^mice has been shown to require the proapoptotic gene *Bax*, indicating that these neurons undergo apoptosis in the absence of BDNF [67]. BDNF probably functions to block cell death by a similar mechanism in geniculate neurons, although the geniculate has not yet been examined in double *Bdnf*^-/-^*/Bax*^-/-^mice. It is also not yet clear which cells types are dying in BDNF (i.e. differentiated neurons or neuronal precursors). The specific role(s) of NT4/5 is even less clear. The earlier loss of geniculate neurons in *Ntf5*^-/- ^mice compared with *Bdnf*^-/- ^mice, could be due to NT4/5 regulation of proliferation or exit from the cell cycle. Also, a very different population of cells may be regulated early in development than is affected later in development by removal of NT4/5. Neither the timing nor the specific role of NT3 for geniculate neurons, have been examined.

It is worth mentioning that growth factors other than the neurotrophins may regulate geniculate neuron cell cycle events. For example, geniculate neurons express receptors for members of the GDNF family [68]. Although it is not clear whether these factors influence geniculate neuron development, removal of GDNF does reduce the number of neurons in the nodose/petrosal ganglion [69]. It remains unclear, however, whether any of these neurons innervate taste buds. Finally, one cannot ignore the possibility that all of the growth factors discussed so far may regulate neuron survival but not proliferation. If this is the case, then it is not known what factors may mediate developmental changes in proliferation in the gustatory ganglia.

### Gustatory axon outgrowth and guidance

Neurite extension occurs as neuronal precursors become post-mitotic and differentiate. *In vitro*, geniculate axon outgrowth requires the addition of a neurotrophin to the culture media. BDNF, NT4/5, and GDNF are capable of supporting neurite outgrowth, while NT3 and NGF are not [70]. Interestingly, the removal of BDNF or NT4/5 does not disrupt the ability of chorda tympani axons to reach the tongue [71]. These findings indicate that while a neurotrophin is required for geniculate axon outgrowth, neurotrophins are capable functioning redundantly *in vivo *to support the growth of axons.

Axons of the chorda tympani must navigate the distance from the geniculate ganglion to the lingual epithelium of the dorsal tongue. These axons grow into the tongue as it develops (E12) [33,72] and approach the epithelial surface by E13.5. Because chorda tympani axons follow precise, spatially restricted pathways to the tongue surface, a series of molecular cues from the environment must guide these axons to the lingual epithelium [73]. It is likely that multiple attractive and repulsive cues are required to ensure that gustatory axons maintain the proper path from the ganglion to the lingual epithelium [74].

Multiple families of well-established axon guidance cues [75] exist and include the netrins, slits, semaphorins, and ephrins. While any of these factors may regulate axon guidance in gustatory neurons, most remain un-investigated in the taste system. One exception is the chemorepulsive factor, semaphorin 3A (Sema3A). Sema3A is expressed in developing tongue [76] and appears to be important during both trigeminal and chorda tympani axon guidance [70,72]. Sema3A expression decreases from the medial to lateral tongue surface and prevents premature and aberrant growth of trigeminal and gustatory fibers into the tongue mid-region. In addition, as geniculate axons near the epithelial surface, Sema3A prevents premature penetration of the epithelium [77,78]. Another member of this family, Sema3F, is also expressed by lingual epithelium, although its function remains unclear [78].

While semaphorins may be the primary chemorepellent molecules used by chorda tympani axons, multiple chemoattractants are undoubtedly also required to guide chorda tympani axons to fungiform papillae. For example, factors produced by the tongue may encourage initial tongue innervation. Other factors produced by the lingual epithelium could attract chorda tympani fibers to the dorsal epithelial surface. Consistent with the idea that the tongue contains chemoattractants, the axolotl oropharyngeal endoderm, which gives rise to taste buds, is chemoattractive for early gustatory neurons [79]. Flank ectoderm is also initially chemoattractive. While pharyngeal ectoderm retains its ability to attract gustatory neurons during taste system development, the flank ectoderm loses its attractive qualities. Perhaps gene expression profiling of these two tissues across different developmental time points will enable the identification of important chemoattractant factors. Not surprisingly, the mammalian tongue is also chemoattractive [78]; however, virtually nothing is known about the factor responsible for its chemoattractive properties or if it has any similarity to the amphibian tongue chemoattractant. *In vitro *experiments reveal that the chemoattractive properties of the mammalian tongue are not affected by the presence of BDNF or NT4/5, ruling out the possibility that the chemoattractant is one of these neurotrophins [78]. Obviously, isolating the tongue chemoattractant factor(s) is an important next step in determining how gustatory axons navigate from the gustatory ganglia into the base of the tongue and toward the lingual epithelium. Future experiments should also address whether the soft palate is as chemoattractive as the tongue for geniculate neurons and whether the tongue is chemoattractive for petrosal neurons.

The identification and functional evaluation of the guidance cues important for the taste system may be complicated by the possibility that these cues may interact with other environmental factors, like neurotrophins, to produce a unique effect. For instance, NT4/5, but not BDNF, enhances the responses of geniculate neurons to Sema3A and Sema3F [78]. In mice that over express NT4/5 in the epithelium, chorda tympani fibers remain below the surface as if repelled by the lingual epithelium [80]. Because Sema3A normally inhibits chorda tympani innervation of the lingual epithelium [77], NT4/5 repulsion could be explained, at least in part, by NT4/5-mediated enhancement of geniculate fiber responses to Sema3A. NGF, on the other hand, has been shown to reduce the sensitivity of somatosensory neurons to Sema3A [77,81]. BDNF may act similarly by reducing the sensitivity of geniculate neurons to Sema3A inhibition, although it has not been shown to inhibit responses to Sema3A in culture [78].

### Summary of geniculate and petrosal ganglion development

The geniculate and petrosal ganglia arise primarily from epibranchial placodes, which arise from a common placodal region. Epibranchial placode formation appears to be regulated by a series of transcription factors and signals from the pharyngeal pouch endoderm, like BMP7 (Figure 2). *Six1 *and *Eya1 *regulate specification of the pan-placodal region, and *Pax2 *may confer additional specification upon this region to produce the epibranchial and otic placodes. *Ngn2*, *Phox2a*, and *Phox2b *are all important regulators of neuronal differentiation in the geniculate and petrosal ganglia. The identity of the factors that regulate the gustatory neuron phenotype and subtypes is unknown. Likewise, it is unclear what factors may regulate neuroblast proliferation in either the geniculate or the petrosal ganglion. However, the neurotrophins BDNF, NT4/5, and perhaps NT3 are important for the survival of differentiated gustatory neurons and possibly of neuronal precursors. Neurotrophins are also required for axon outgrowth from the geniculate ganglion; however, different neurotrophic factors function redundantly such that no single neurotrophin is required. In general, very little is known about the factors important for gustatory axon guidance. While semaphorins function as important chemorepellents during gustatory axon guidance, no chemoattractants have been isolated. In addition, it is not known whether semaphorins regulate axon guidance for petrosal gustatory neurons innervating the circumvallate papillae.

## Development and innervation of gustatory papillae and taste buds

### Taste papilla formation

Taste buds on the tongue are located in three specialized types of epithelial structures called papillae. In mice, approximately 90 fungiform papillae (180 in rats) occupy the rostral two-thirds of the tongue, and each typically contains one taste bud. A single circumvallate papilla is located on the midline of the caudal tongue, and folds (foliate papillae) are located at the lateral edges of the caudal tongue. When the tongue initially forms, around E12 in mouse, the surface is homogenous. Placodal thickenings then arise on the surface by E13. At E13, fungiform placodal thickenings (or placodes) occupy two bilateral rows adjacent to the mid-line; however, by E14.5 a full complement of developing fungiform papillae is present on the tongue [32]. The single circumvallate papilla, which will house taste buds on the back of the tongue, also arises at E13 as a swelling on the midline of the back of the tongue [82]. As the development of fungiform papillae proceeds, the placodal edges extend into the underlying mesenchyme and evaginate into a raised structure. Once the basic papillae shape is established, papillae continue to differentiate. The epithelial cells (keratinocytes) at the papillary surface, for example, become squamous. On the soft palate, taste buds are not located in papillae; however, they are located in slightly raised areas (eminences) on the palatal surface. The majority of taste buds are in two long eminences, which overlie the regions of the tongue just lateral to the circumvallate papillae; these regions are the geschmacksstreifen (taste stripe) [83].

Signaling factors involved in epithelial patterning in numerous different tissues are also expressed on the tongue surface during development. These factors include sonic hedge hog (Shh), the bone morphogenic proteins (Bmp 2, 4), Noggin [84], fibroblast growth factor 8 (FGF 8) [85-87], Sox-2 [88], and Wnt ligands [88]. Recently, there has been considerable progress in the understanding of how some of these factors regulate papilla morphogenesis. Because this work has been recently reviewed [89], I will only touch on it briefly here.

Wnts, a large ligand family, function via multiple receptor mediated pathways, one of which involves β-catenin activation which results in transcriptional activation of Lef1 and Tcf transcription factors. Activation of this pathway by addition of LiCl to tongue cultures increases placode number [90,91]. Disruption of β-catenin signaling, by either genetic deletion of epithelial β-catenin, Lef1, Wnt10b, or overexpression of a β-catenin antagonist (dickkopf1), blocks fungiform placode development [90,91]. A dominate stabilizing mutation of β-catenin causes a dramatic increase in papillae number, such that the tongue is completely covered by fungiform papillae [90]. Wnt-β-catenin signalling regulates Sox-2 expression [88]. When Sox2 expression is reduce to 20% of normal levels fungiform placodes form, but papillae fail to develop to maturity. Overexpression of Sox2 increases fungiform papillae at the expense of filiform papillae development.

In addition to regulating Sox-2, Wnt-β-catenin signaling interacts with Shh signaling [91], although the nature of this interaction is unclear. Although Shh knockout mice lack tongues, Shh can be functionally manipulated in tongue organ cultures using the steroidal plant alkaloids cyclopamine or jervine, or a function-blocking anti-Shh antibody [92-94]. Disruption of Shh signaling, following initial tongue development, increases the number and size of fungiform papillae on the tongue surface [92-94]. The normal pattern of fungiform papillae is also disrupted, and many fungiform papillae become fused. Pre-incubation of tongue cultures with epidermal growth factor (EGF) blocks the effects of cyclopamine on papillary number [95]. EGF is expressed in inter-papillary regions [93], and when added to tongue cultures, it inhibits papilla morphogenesis in a dose-dependent manner [95]. Thus, it may be that EGF and Shh interact with one another to regulate papillary number.

Lastly, fungiform placode development can also be inhibited by multiple BMPs. When these proteins are delivered by attaching the protein to a bead that is inserted just beneath the lingual surface, or directly in the culture medium of the E14 rat tongue, fungiform placodes fail to form the region of the bead [84]. In contrast, the BMP antagonist, noggin, can increase the number of placodes of the tongue surface [84]. Therefore, these factors may function together to regulate the distribution of fungiform placodes on the tongue surface. Taken together these results indicate that a large number of patterning factors function together to orchestrate the developmental pattern of fungiform papillae on the tongue surface. Much less clear are the mechanisms by which these factors function and whether or not they function upstream or downstream of each other. It is likely that future studies will focus on clarifying these issues.

### Targeting of gustatory axons to fungiform papillae

**Figure 5:**
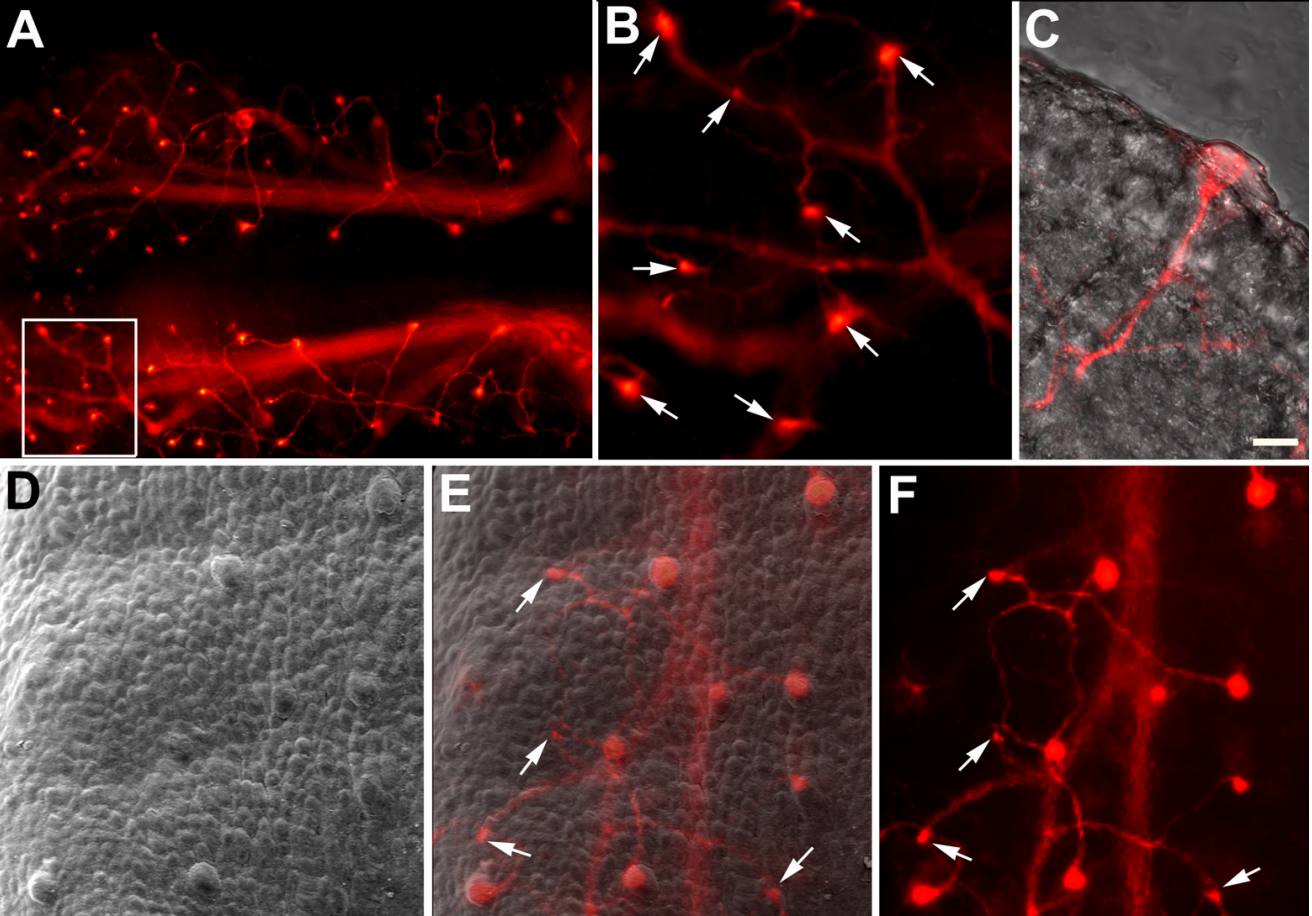
**By E14.5, chorda tympani fibers have innervated fungiform papillae.** Chorda tympani fibers have a stereotypical branching pattern in the tongue (A). A higher magnification view of the area outlined in (A) illustrates that each fiber bundle ends in a distinctive bulb shape known as a neural bud (B, arrows). Neural buds form when gustatory fibers penetrate the epithelium at the surface of a fungiform papilla (C). All papillae appear to be innervated when the image from a portion of a E16.5 tongue following DiI-labeling (D) and the SEM image from the same tongue region (F) are overlaid (E). However, there are some neural buds in locations where no fungiform papillae are present (arrows in E and F).

Fungiform placodes/papillae are organized in a very stereotyped array on the tongue surface [96]. Chorda tympani fibers provide innervation to these locations while the lingual branch of the trigeminal innervates all adjacent epithelia. Chorda tympani fibers innervate fungiform papillae on most of the dorsal tongue surface on E14.5 of development. When fibers penetrate the epithelium they form a distinctive ending called a neural bud (Figure 5) [32]. To examine the accuracy of the initial innervation to fungiform papillae, our laboratory has developed new anatomical approach that combines DiI-labeling with Scanning Electron Microscopy (SEM). Neural buds can be identified on the tongue surface via DiI-labeling, and fungiform placodes/papillae can be independently identified in the same tongue using SEM [32]. By overlaying DiI-labeled and SEM images of the same tongue, we were able to determine whether each fungiform placode was successfully innervated. Our results revealed that at E14.5, immediately following the initial target innervation, most chorda tympani fiber bundles reach the correct location. Some errors in targeting do occur; a few fungiform papillae are not initially innervated, and there are regions on the tongue receiving innervation where no fungiform papilla is present. A post-targeting refinement of innervation improves upon the accuracy of the initial targeting.

**Figure 6:**
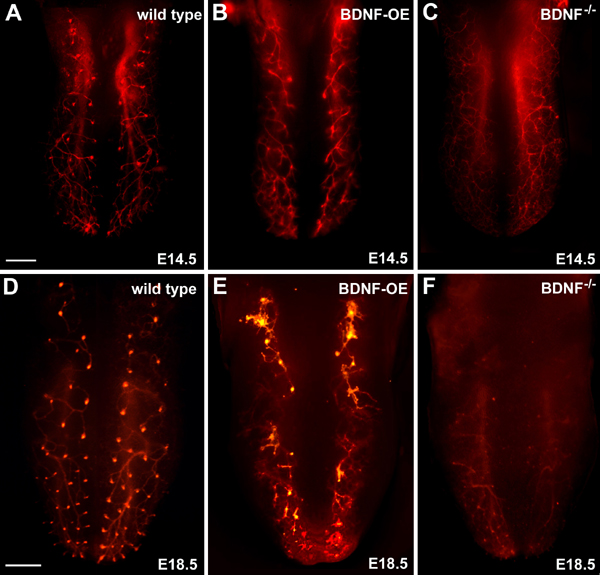
**DiI-labeling reveals that normal innervation patterns (A, D) are disrupted by overexpression of BDNF (B, E) and loss of BDNF (C, F)**. By the first day of targeting (E14.5), wild type mice have very stereotyped innervation patterns, and each fiber bundle branch ends in a neural bud (A). Overexpression of BDNF throughout the epithelium increases branching and disrupts normal targeting (B, E). Very few neural buds are initially formed (B) and by E18.5 (E), few fungiform papillae are innervated. Innervation patterns are even more disrupted in *Bdnf*^-/- ^mice. Axonal branching is extensive near the epithelial surface and neural buds fail to form (C). By E18.5, there is a loss of innervation throughout most of the tongue; however, a few neural buds were present, indicating that some fungiform papillae were innervated. Scale bar in A = 250 μm, applies to A-C; Scale bar in D = 500 μm, applies to D-F.

The fact that initial targeting is very accurate suggests that some factor must be expressed in the developing fungiform papillae to signal the correct location to innervating chorda tympani axons. Converging evidence indicates that BDNF functions in this capacity. BDNF is produced by developing fungiform papillae before they are innervated [55,56,97] and has been shown to function as a chemoattractant for developing chorda tympani fiber bundles *in vitro *[98]. In addition, BDNF is capable of attracting chorda tympani fibers to innervate inappropriate locations *in vivo*. When BDNF is over expressed throughout the entire epithelium under control of a keratin 14 promoter (BDNF-OE), chorda tympani innervation patterns are disrupted (Figure 6B,E), and chorda tympani fibers fail to innervate most fungiform papillae [99]. A similar failure of gustatory fibers to innervate fungiform papillae occurs when BDNF is over expressed in muscle and within the ganglion itself [100]. A detailed analysis of altered innervation patterns in BDNF-OE mice demonstrated that chorda tympani fibers were attracted to and invaded non-taste filiform papillae instead of gustatory papillae [80]. This finding demonstrates that BDNF expressed in non-taste papillae can attract chorda tympani fibers to these regions and cause them to become innervated.

To determine if BDNF is required for normal target innervation, we recently examined target innervation in mice lacking BDNF [71]. At E14.5 and E16.5, these animals had chorda tympani fibers that branched extensively below the epithelium, but did not penetrate the epithelium and form a neural bud (Figure 6C,D). This increased branching occurred despite the fact that these mice were losing geniculate neurons between E14.5 and E16.5. Although target innervation is disrupted for some other BDNF-dependent sensory systems in *Bdnf*^-/- ^mice [67,101], this is the first demonstration of increased branching in response to BDNF removal. It is as if in the absence of BDNF gustatory fibers are hyperinnervating the tongue in order to find their targets. Eventually, four days after neural buds normally form (E18.5); a few fungiform papillae are innervated in *Bdnf*^-/- ^mice. We also examined the distribution of innervation to the soft palate, where targeting is not preceded by papilla formation. *Bdnf*^-/- ^mice displayed increased branching of nerve fibers in the soft palate and a loss of specific innervation to the taste bud containing areas. Therefore, BDNF is required for geniculate neurons to innervate gustatory epithelia successfully, even in the soft palate where gustatory papillae are not present. A few neural buds did finally form at E17.5, three days after they appear in wild type mice. These targeting effects are specific to *Bdnf*^-/-^mice and do not occur in *Ntf5*^-/- ^mice, even through NT4/5 binds to the same receptors as BDNF. Taken together, these findings demonstrate that BDNF functions as a short range chemoattractant which allows facial gustatory neurons to locate and innervate taste epithelia during development.

### Taste bud development

**Figure 7:**
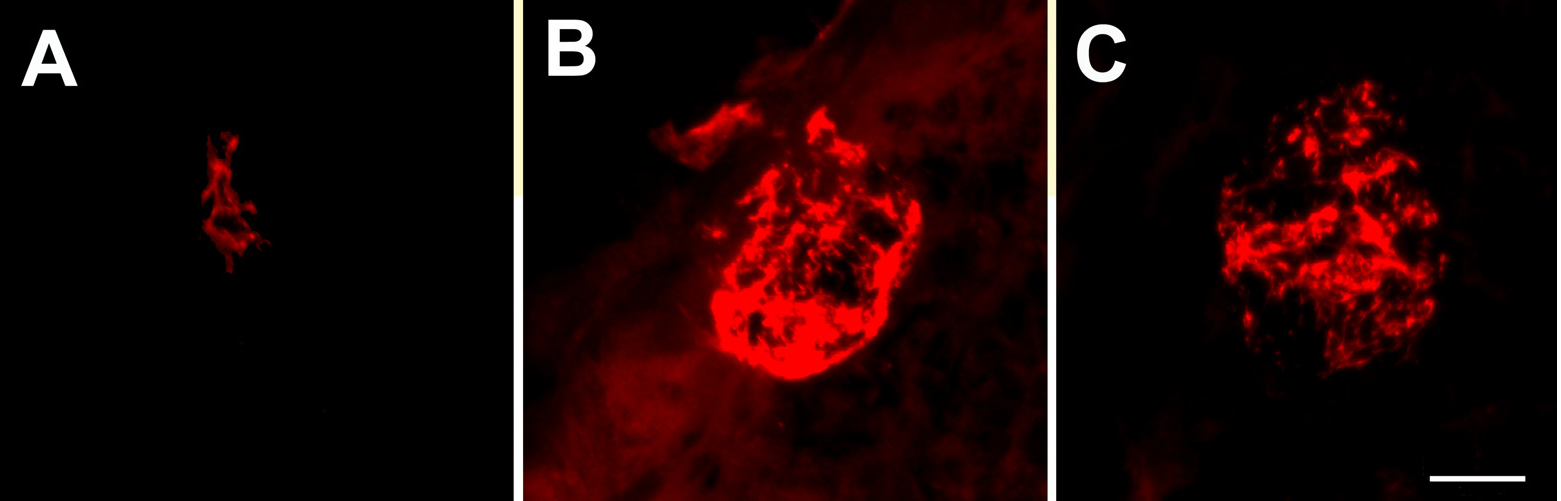
**Small groups of taste cells are labeled with anti-troma I (anti-keratin 8) as early as E16.5 (A).** These clusters increase in size and number by E18.5 (B) and by birth (C). Scale bar in F = 10 μm, applies to A-C.

Taste buds arise from oral epithelium [102-104]. The timing of their initial development is under debate. Using the intermediate filament cytokeratin 8 as a marker for taste buds, taste bud precursors have been observed as early as E13.5 in mice [105]. However, keratin 8 is also present in the flattened layer of periderm, which forms as the embryonic epithelium becomes bi-layered [[106]317]. Our laboratory, as well as others [8], have had a difficult time distinguishing these taste placodes from the brightly labeled periderm at this early age. In the mouse, a small number (10 ± 7) of brightly labeled groups of keratin 8 positive cells are observed clearly within fungiform papillae by E16.5 (Figure 7A). These cell clusters become much larger and increase in number by E18.5 (Figure 7B), but do not adopt an adult-like morphology until well after birth. Thus, we do not observe keratin 8 positive taste buds until well after taste fibers penetrate the epithelial surface (E14.5). The morphological differentiation of taste cells also occurs as or after nerve fibers penetrate the epithelium [34,107]. The fact that innervation precedes the differentiation of taste buds does not indicate that gustatory fibers induce taste bud formation. Although this is one possibility, factors from tissues other than nerve fibers likely influence initial taste bud development.

In amphibians, epithelial cell-cell interactions induce taste bud formation [108], indicating the epithelia from which taste buds differentiate contain at least one signaling factor for taste bud induction. It is also possible that epithelial cell-cell interactions are important for the early induction of taste buds in mammals, long before taste buds differentiate [1,3]. Following the initial induction, taste bud differentiation may occur in response to a different signal, perhaps from nerve fibers [46]. At this point, there is no experimental evidence either supporting or refuting this possibility. Experimental evidence is lacking primarily because there are no markers for mammalian taste progenitors. Without such markers, it is impossible to determine when the taste bud is first induced. Fate mapping experiments that clearly identify the population of cells that is destined to become a taste bud will be required to address this issue. Currently, taste buds can be observed only once they differentiate. We do know that, in mammals, lingual taste buds can form only within papillae, which indicates that taste bud-competent cells exist before taste buds differentiate and are restricted to gustatory papillae. It is possible that these cells become specified as gustatory placodes initially develop.

Following the initial induction and early differentiation of taste buds, some synapses between taste cells and nerve fibers can be seen [109]. The very last feature of the taste bud to develop is the taste pore, a small hole in the keratinized epithelia of the papilla that allows solutions to access the sensory cells. Taste pores appear slowly during postnatal development [110]. Adult taste buds are complex sensory organs with multiple cell types including supporting cells, cells capable of sensory transduction, and cells with synaptic connections to neurons [111,112]. It is reasonable that multiple factors would be involved in the differentiation of a receptor end organ with such complexity. While the factors controlling taste system development are still unknown, multiple candidates have been identified based on their roles in other animals/systems and their expression patterns in taste buds. These factors include Shh and Nkx2.2, a factor that functions downstream of Shh and is present in some taste cells [85,93,113]. Transcription factors related to the Drosophila *aschaete-schute *complex may regulate taste bud development, as in gustatory ganglion development [113-118]. Growth factors functioning via tyrosine kinase receptors may regulate taste bud development and/or trophic maintenance of taste buds [119]. Unfortunately, the specific roles of each of these factors in regulating taste bud development cannot be determined until multiple approaches are developed that allow for selective gene removal from developing and adult taste cells.

### Summary of peripheral gustatory development

Lingual taste buds develop within specialized papillae. Multiple signaling factors are expressed by the developing placodes that will become papillae. These factors regulate the formation, size and pattern of gustatory placodes. Gustatory epithelium in the soft palate is not defined by specialized papillae. Future experiments should address whether these same factors have a similar expression pattern and role for taste bud-containing regions on the soft palate. After fungiform papillae are formed, they become innervated. BDNF expressed within fungiform papillae regulates target innervation and appears to be the primary factor that allows gustatory axons to distinguish taste from non-taste epithelia. BDNF also appears to be important for the targeting of geniculate neurons to the soft palate. However, it is not known whether gustatory neurons of the petrosal ganglion require BDNF to correctly locate circumvallate or foliate papillae. It is also not known whether BDNF is regulated downstream of factors that control fungiform placode development. The mechanisms by which taste buds are induced in mammals and the factors regulating their differentiation are still fundamentally unclear. Several transcription factors are expressed in taste buds of the circumvallate papilla and could be important for taste bud development. Further characterization of the localization of each of these factors in taste bud-containing regions could provide clues as to their specific roles in taste bud development.

## Development of gustatory axon innervation to the central nervous system

In addition to innervating taste buds during development, primary gustatory neurons project to specific locations in the CNS. Central branches of gustatory axons from the geniculate and petrosal ganglia terminate in the rostral portion of the nucleus of the solitary tract (NST). The terminal field for the petrosal neurons is caudal to that of the geniculate neurons, although some overlap exists between these fields. The NST serves as an integration center for visceral sensory input to the brainstem. In rat, axons of the geniculate ganglion begin to invade the NST at E15 [120]. By E17, geniculate terminal fields ramify in the rostral NST, and the general morphology is adult-like by E19. However, the terminal field of the chorda tympani nerve continues to increase in size through postnatal day 15 (PN15), and extensive remodeling occurs up to PN25 [121,122]. The first synaptic thickenings in the rostral NST are detectable at E17, and the first synaptic vesicles are observed at E19 [120].

### Outgrowth, guidance, and terminal field formation of CNS projections

A careful examination of the factors regulating the outgrowth and guidance of central gustatory axons is lacking. It is reasonable to speculate that some of the same factors that influence peripheral axon outgrowth and guidance may also be important centrally. Sema3A, for example, may function as an inhibitory molecular cue for central axon guidance, much as it does in the periphery. It is also possible that neurotrophins are important for CNS process outgrowth from the geniculate ganglion.

The ability of BDNF to regulate peripheral targeting may point to a role for this neurotrophin in the development of central targeting and terminal field innervation. Clues as to how central targeting is regulated by neurotrophins in the taste system may be gleaned from studies examining the role of neurotrophins in CNS targeting in other sensory neurons. For instance, the elimination of the pro-apoptotic BCL-2 homolog, Bax, allows sensory neurons survive in the absence of neurotrophins [123-125]. Although NT3-dependent proprioreceptive neurons survive under these conditions, they do not project far enough into the spinal cord to reach the correct location for the formation of a terminal field [126]. This deficit is attributable to the failure of these neurons to express the transcription factor ER81, which is required for central axons to reach their targets. These data reveal that NT3 mediates the formation of proprioceptive afferent-motor neuron connections via regulation of ER81. Interestingly, not all sensory neurons require neurotrophins for CNS targeting. Sensory neurons (nociceptors) that require NGF/trkA signaling for establishing and maintaining cutaneous innervation during development do not require NGF for central targeting [127]. Thus, it is yet to be determined whether BDNF-dependent gustatory neurons reach the NST in the absence of functional BDNF.

While very little is known about the effects of growth factors or transcription factors on terminal field development, it is clear that environmental manipulations during embryonic development influence the eventual size of the terminal field. As an example, restriction of dietary sodium during a brief embryonic period from E3 to E12 results in an enlarged gustatory terminal field by adulthood [128,129]. This critical period for dietary effects on terminal field development occurs before chorda tympani axons reach the taste bud or the NST. This effect is not specific for sodium deprivation; protein deprivation during development can also result in enlarged gustatory terminal fields (Dr David L. Hill, personal communication). Therefore, this effect could be the result of general nutritional deficits. One possible explanation for these findings is that early dietary deprivation disrupts or enhances the developmental expression of some factor(s), like growth factors, which in turn regulate gustatory terminal field formation later in development.

### Development of post-synaptic neurons in the NST

A number of studies have examined the morphological changes in postsynaptic gustatory neurons in the NST during development, but far fewer studies have examined the factors that regulate the development of these neurons. These studies reveal that some of the same factors that regulate primary sensory neuron differentiation also regulate neuronal differentiation in the rostral NST. An example is *Phox2b*. This transcription factor is expressed by NST neurons and neuronal precursors [21,130] and the NST does not form in its absence [21]. Since the gustatory ganglia degenerate in *Phox2b*^-/- ^mice, *Phox2b *may function as a "circuit-specific" transcription factor that helps coordinate visceral sensory circuit formation.

In addition to *Phox2b*, neurons of the NST also express another homeobox gene, *Tlx3 *[131]. In the absence of *Tlx3*, the *Phox2b*-positive neurons that will eventually form the NTS are born but are lost by E12.5. It is interesting to note that, later in development, *Tlx3 *expression is lost from many brainstem areas, but high expression is maintained in the rostral (gustatory) NTS, suggesting that Tlx3 may play a role in the later differentiation of neurons. In dorsal horn of the spinal cord, Tlx3 selects a glutamatergic over a GABAergic cell fate for the postsynaptic neurons receiving sensory information [132,133]. It is possible that Tlx3 also controls the neurotransmitter fate of gustatory postsynaptic neurons in the rostral NST. Taken together, these data indicate the same set of transcriptional regulators may be required for the formation and continued differentiation of multiple components of the gustatory neuronal network. Unfortunately, it is unclear whether these transcription factors are as important for gustatory development as they are for the development of other chemoreceptors [21,25,134-136].

### Summary of gustatory NST development

Almost nothing is known about the factors regulating central projections of gustatory axons. However, it is possible that some of the same factors that regulate peripheral axon development also influence CNS terminal field development. For example, BDNF could influence the ability of CNS gustatory neurons to terminate correctly in the NST, much as it is influences targeting in the periphery. Likewise, the transcription factors that regulate development of the geniculate and petrosal ganglia may also regulate the development of postsynaptic neurons in the NST. For example, the transcription factor *Phox2b*, which regulates gustatory ganglion differentiation, is required for the differentiation of NST neurons. The transcription factor Tlx3 is also expressed by rostral NST, and it may regulate the development of specific NST neuron phenotypes.

## Conclusion

The formation of gustatory system requires a complex set of processes that are regulated by a number of different molecular cues. In recent years we have learned a fair amount about the molecular cues regulating early gustatory differentiation, neuron cell survival, gustatory papilla development and peripheral targeting. However, the specific cellular mechanisms used by these factors to regulate gustatory development are still unknown. Almost nothing is known about the factors regulating the development of gustatory neuroblast proliferation, specific gustatory neuron phenotypes, gustatory axon guidance, taste bud induction/differentiation and CNS targeting. Determining what these factors are and the mechanisms by which they function is important not only for a better understand of gustatory development, but developmental neurobiology in general.

## Competing interests

The authors declare that they have no competing interests.

## Authors' contributions

This manuscript was written by RFK.
